# Epstein–Barr virus-associated hemophagocytic lymphohistiocytosis in a small child: A case report

**DOI:** 10.1097/MD.0000000000018759

**Published:** 2020-01-17

**Authors:** Maria Oana Mărginean, Eniko Molnar, Mihaela Ioana Chinceşan

**Affiliations:** a Department of Pediatrics, “George Emil Palade” University of Medicine, Pharmacy, Sciences and Technology of Târgu Mureş, Romania; b Department of Laboratory Medicine, County Emergency Clinical Hospital of Târgu Mureş, Târgu Mureş, Romania.

**Keywords:** child, epistaxis, Epstein–Barr virus, hemophagocytic lymphohistiocytosis, macrophage activation, thrombocytopenia

## Abstract

**Introduction:**

Hemophagocytic lymphohistiocytosis (HLH) is a rare, potentially lethal disorder, characterized by a dysregulation of the immune response, leading to a severe inflammatory syndrome. Epstein–Barr virus (EBV)-associated HLH is a form of secondary HLH, a fulminant presentation of an otherwise benign viral infection.

**Patient concerns:**

We report the case of a 3-year-old girl who presented with fever, signs of accute upper respiratory tract infection and spontaneous, disseminated ecchymoses. Initial laboratory tests revealed pancytopenia. A bone marrow aspirate was performed, which revealed megaloblasts and numerous macrophages, with abundant foamy cytoplasm. Megaloblastic anemia was excluded, as the levels of vitamin B12 and folic acid were both within normal ranges.

**Diagnosis.:**

Hyperferritinemia, hypertriglyceridemia, hypofibrinogenemia, and splenomegaly were relevant criteria for the diagnosis of HLH, in accordance with the bone marrow specimen. Positive immunoglobulin M antibodies for EBV were supportive of an acute EBV infection, which was the most probable trigger of HLH. The patient's evolution was complicated by a massive epistaxis, in the context of thrombocytopenia which required plasma, thrombocyte, and erythrocyte substitutes.

**Intervention.:**

The patient was started on a treatment regimen of 8 weeks with etoposide and dexamethasone.

**Outcome:**

Her evolution was favorable, the treatment being successful in remission induction.

**Conclusion:**

Our case emphasizes the diagnostic challenges of HLH, in a patient with EBV infection whose evolution was hindered by a severe epistaxis, with potentially fatal outcome.

## 1 Introduction

Hemophagocytic lymphohistiocytosis (HLH) is a condition with potentially life-threatening complications, caused by a dysfunctional immune response, which leads to a severe inflammatory syndrome.^[1,2]^ HLH has been divided into 2 categories: primary and secondary HLH. Primary or familial HLH (FHLH) is a rare disorder, resulting from various genetic mutations.^[3]^ An autosomal recessive disorder, it has been classified into 5 different types, ranging from FHLH-1 to FHLH-5.^[4]^ Secondary HLH, also termed in the past as virus-associated hemophagocytic syndrome (VAHS) and/or malignancy-associated hemophagocytic syndrome (MAHS),^[3]^ is usually triggered by an infection, autoimmune disorder or malignancy in subjects without known genetic abnormalities.^[5]^ However, recent literature data describe a genetic susceptibility in all patients presenting with HLH.^[6]^ Macrophage activation syndrome (MAS), an entity belonging to secondary forms of HLH, is a term used mostly in association with pediatric rheumatic diseases, such as systemic-onset juvenile rheumatoid arthritis, Kawasaki disease, or systemic lupus erythematosus.^[2,3]^

The pathogenesis of FHLH involves a hyperactivity of CD 8 + T lymphocytes and macrophages due to an impairment of cytotoxic T cell and natural killer (NK) cell function. The latter play a key role in modulating the immune response, by inhibiting the activation of antigen-specific T cells. Overwhelming activation of macrophages and T cells leads to an increased expression of proinflammatory cytokines, with excessive circulatory levels leading to organ dysfunction and hematologic abnormalities.^[7,8]^ In particular, Epstein–Barr virus (EBV), through its ability of activating CD 8 + T lymphocytes, can cause a hyperproduction of interferon-gamma (IFNγ), which can trigger antigen-presenting cells.^[9]^

HLH typically presents with persistent fever, hepatosplenomegaly, and pancytopenia. Although the clinical tableau is very important in establishing a diagnosis, most professionals prefer to refer to the HLH-2004 diagnostic criteria before drawing a conclusion.^[10]^ Besides the presence of fever, splenomegaly, and cytopenia (at least two cellular lines in the peripheral blood), there are other criteria relevant for the diagnosis: hypertriglyceridemia and/or hypofibrinogenemia, hemophagocytosis in bone marrow or spleen or lymph nodes, lack of evidence to support the presence of a malignancy, hyperferritinemia, low/absent activity of NK cells, and high levels of the interleukin 2 soluble receptor.^[10]^ Five of 8 criteria need to be met for the diagnosis of HLH, unless a molecular diagnosis of HLH can be established. However, the HLH-2004 guideline underlines the importance of searching for a proof of hemophagocytosis, starting from a bone marrow aspirate.^[10]^ A new score has been developed for the diagnosis of reactive HLH in 2014 by the American College of Rheumatology, based on the HLH-2004 criteria, with the addition of liver involvement among relevant diagnostic elements.^[11]^ The H-score is obtained depending on the severity of each of the clinical and paraclinical parameters evaluated. This score then translates into the individual probability of having secondary HLH.^[11]^ Developed only for adult populations, this score can be a useful tool in pediatric HLH as well, with a better diagnostic accuracy than the classical HLH-2004 criteria, according to a Belgian study.^[12]^

HLH can be fatal in the absence of early specific treatment. The HLH-2004 treatment protocol has proven its efficacy, with reported 5-year survival rates as high as 66%.^[13]^ This has been a major breakthrough, considering the poor prognosis of FHLH in the past, with 1-year-survival rate of <5% from the time of the diagnosis.^[8]^ Although FHLH requires hematopoietic cell transplantation as definitive cure,^[8]^ complete treatment of secondary HLH can be achieved with the help of the standard HLH-2004 regimen. However, the medication protocol needs to be adapted depending on its cause; a special attention must be given to an underlying sepsis, lymphoma, or leukemia.^[14,15]^ Consistent follow-up of all remisive cases of HLH is required.^[14]^

This case report aims to underline the diagnostic challenges of HLH, as well as its potentially lethal, hemorrhagic complications.

The written informed consent was obtained from the patient's mother before publication of this case.

## 2 Case report

### 2.1 Presenting concerns

A previously healthy 3-year-old female patient presented to a local hospital with fever, signs of acute upper respiratory tract infection, and multiple, disseminated ecchymoses on the trunk and limbs in the absence of recent trauma. The laboratory tests revealed pancytopenia, with severe thrombocytopenia (15,000 cells/μL). The suspicion of a malignant hemopathy was raised. Therefore, the patient was referred to the pediatric hemato-oncology department of a territorial hospital.

### 2.2 Clinical findings

The clinical examination at the time of admission revealed fever, upper respiratory infection, and ecchymoses on the limbs and trunk.

### 2.3 Diagnostic focus and assessment

**Figure 1:**
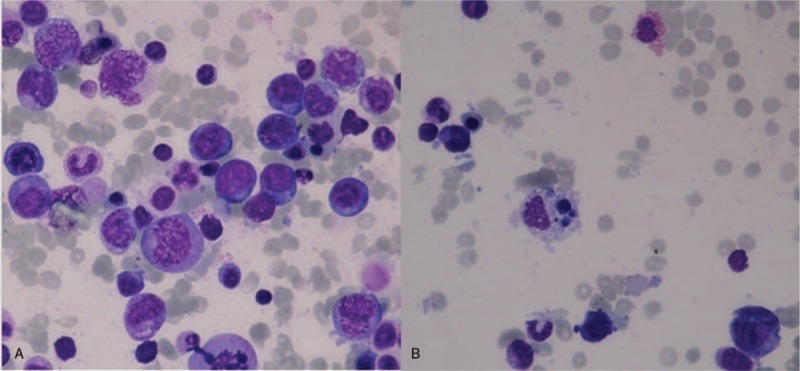
(A) Dysplasia of the erythroblastic cell line, megaloblasts with sporadic karyorrhexis, and Howell-Jolly bodies, accompanied by numerous macrophages (100× magnification). (B) Macrophage with abundant foamy cytoplasm (100× magnification).

The laboratory tests performed at the time of admission in our service revealed normal leukocyte count (4430 cells/μL) with neutropenia (870 cells/μL), anemia (erythrocytes: 2.59 × 10^6^ cells/μL, hemoglobin 7.0 g/d\L), and thrombocytopenia (16,000 cells/μL), accompanied by a slightly prolonged prothrombin time (14.6 s). Other relevant initial data included increased aspartate aminotransferase level (40 U/L) and erythrocyte sedimentation rate (23 mm/h), together with an abnormal high level of lactate dehydrogenase (1214 U/l). An abdominal ultrasound was performed, with unremarkable findings. The peripheral blood smear showed no sign of atypical cells. The bone marrow aspirate described dysplasia of the erythroblastic cell line, accompanied by megaloblasts with sporadic karyorrhexis and Howell-Jolly bodies, as well as the presence of numerous macrophages, with abundant foamy cytoplasm (Fig. 1).

Normal levels of vitamin B12 and folic acid excluded a diagnosis of megaloblastic anemia. As hemophagocytic phenomena were detected in the bone marrow, further investigations were conducted. Total levels of immunoglobulin (Ig) M (197 mg/dL), G (1052 mg/dL) and A (125 mg/dL) were raised, which explained the simultaneous, positive antibodies for EBV, Cytomegalovirus, *Toxoplasma gondii*, and Rubella, as well as the need to reiterate the viral serological tests. Hyperferritinemia (4001 ng/mL), hypertriglyceridemia (522.6 mg/dL), and low fibrinogen level (86 mg/dL) supported the diagnosis of HLH.

### 2.4 Follow-up and outcome

**Figure 2:**
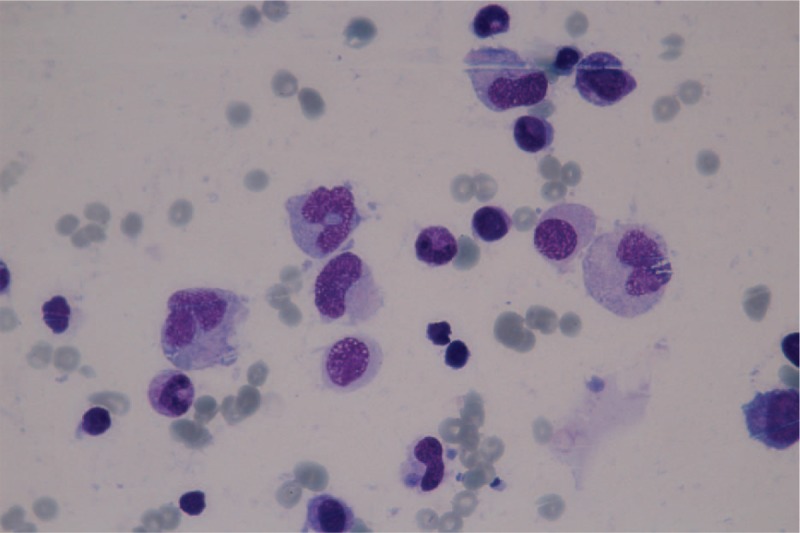
Transformation of monocytes into macrophages (100× magnification).

Continuous, daily thrombocyte substitution provided a slight increase in the platelet level, followed by a decline in their number to the initial value. As a result of the persistent thrombocytopenia, the patient developed a massive epistaxis, which was hardly controlled by anterior nasal packing. The patient was transferred to the pediatric intensive care unit (PICU) in critical state, with severe post-hemorrhagic anemia (hemoglobin 5.16 g/dL). Computed tomography scans of the head and abdomen were performed to exclude other hemorrhagic sites. A slight enlargement of the spleen was the only abnormal finding. During the PICU hospitalization, viral serological tests were once again completed and a recent infection with EBV was documented (IgM: 10.1 U/mL). Antigens for hepatitis B and C were negative, as well as the antibodies for the previously tested viral infections. A second bone marrow specimen depicted transformations of numerous monocytes into macrophages, thus strengthening the diagnosis of HLH (Fig. 2). Supportive treatment with platelet-rich plasma, thrombocyte, and erythrocyte substitutes was successful in stabilizing the patient.

Based on all the clinical and paraclinical data, a diagnosis of secondary HLH, due to an acute EBV infection, was established.

### 2.5 Therapeutic focus and assessment

A treatment regimen of 8 weeks with etoposide and dexamethasone was started, according to the HLH-1994 guidelines. The patient also benefited from cotrimoxazole prophylaxis (5 mg/kg) 3 times a week and gastroprotection with esomeprasole during the therapeutic cycle. The patient's evolution was favorable, with progressive normalization of all cellular lines. The patient is currently under close, periodic follow-up. So far, 4 months after the initial diagnosis, there are no signs of disease reactivation.

## 3 Discussions

The diagnosis of HLH still remains a challenge, due to its nonspecific diagnostic criteria. Its similarity to sepsis is obvious due to some common features, such as fever and leucopenia. Hyperferritinemia (acute phase reactant), low fibrinogen levels, and thrombocytopenia can also be found in sepsis, especially in the context of disseminated intravascular coagulation. An early differential diagnosis of the 2 conditions is mandatory for a proper therapeutic management, as sepsis therapy does not involve any of the chemotherapeutic agents, which can considerably improve survival of HLH.^[16]^ Hematologic malignancies and autoimmune disorders can present clinical similarities to HLH, but can also evolve into HLH. Therefore, a thorough search for an underlying disease must always be conducted.^[17]^ Our patient initially presented with fever and pancytopenia, which were in concordance with various malignant hemopathies and autoimmune diseases. As leukemia and lymphoma were both considered among potential diagnoses, a bone marrow aspiration was performed, which revealed numerous macrophages, but also the presence of an important number of megaloblasts. Pancytopenia can constitute a complication of megaloblastic anemia, which can cause dyserytropoiesis as well.^[18]^ However, this entity was excluded after the dosage of vitamin B12 and folic acid, both within normal ranges. As a result, an HLH diagnosis was suspected and further investigations were conducted.

HLH-2004 guidelines emphasize the need for at least 5 of 8 criteria for a positive diagnosis, as this disorder involves a constellation of symptoms.^[19]^ In our patient's case, we found 6 criteria, which were consistent with a HLH diagnosis: fever, splenomegaly, cytopenia of 3 lineages in the peripheral blood, hypertriglyceridemia and hypofibrinogenemia, evidence of hemophagocytosis in the bone marrow aspirate and hyperferritinemia. IL-2 receptor and NK cell activity could not be performed, as they were not available in our hospital. According to some authors, these 2 paraclinical investigations are not accessible in many hospital settings and the wait for various laboratory results delays the HLH diagnosis. Moreover, they recommend a revision of the HLH-2004 criteria, by introducing clinical and paraclinical assays, which can accelerate the diagnosis.^[17]^

Epstein–Barr virus is considered to be the most frequent infectious factor associated with HLH, especially in Asian populations. Although the incidence of HLH has not been documented in many countries, a national study performed in Japan discovered a frequency of EBV infection as high as 40% among all patients diagnosed with HLH.^[20]^ A higher prevalence in certain geographic areas could be explained by an underlying genetic background. A study performed on a Korean pediatric population documented the presence of a genetic mutation, namely the UNC13D mutation among children with EBV infection who developed HLH, which could partially explain their vulnerability towards exhibiting this immune disorder.^[5]^ Due to its high prevalence in determining HLH, infection with various viral agents, including EBV needed to be searched for. Although the initial serological tests were all positive, the second IgM values were increased only in the case of EBV, which suggested an acute infection. These antibodies explained the particular macrophagic aspect of the monocytes, as visualized in the bone marrow sample.

The H-score, which was more recently developed, seems to have a higher specificity than the HLH-2004 diagnostic guidelines. However, further studies need to be conducted in order to determine the optimal cutoff values.^[21]^ Some authors state that this score is easier to apply into routine practice than the traditional criteria, as it can easily be accessed online (http://saintantoine.aphp.fr/score/).^[22]^ With the help of the same platform, we calculated the score using the parameters of our patient and obtained a probability of 98.89%.

Complications of HLH can endanger life, even with proper, early therapeutic approach. An American study performed on 73 adult patients with HLH described sepsis and multiorgan disfunction as the 2 most frequent causes of death, the 1-year survival rate being only 48%.^[23]^ Another study cites intracranial, gastrointestinal, and diffuse alveolar hemorrhage among one-third of the patients with fatal outcome. These hemorrhagic complications were described in direct relation with the severity of thrombocytopenia and hypofibrinogenemia.^[24]^ Several case reports described severe epistaxis as initial manifestations or aggravations of HLH, in patients with pancytopenia.^[25,26]^ Similarly, our patient developed a serious nasal bleeding, in the circumstances of pancytopenia, hypofibrinogenemia and modified coagulation parameters.

Treatment of HLH comprised of dexamethasone and etoposide, according to the HLH-1994 guideline. The HLH-2004 guidelines recommended the addition of cyclosporine to the protocol, but without any strong evidence to support its benefits. Etoposide seems to be very efficient in EBV-associated HLH, especially by inhibiting the proliferation of the viral nuclear antigen.^[27]^ Due to all these findings, we opted for the HLH-1994 treatment protocol in the case of our patient, which proved to be successful in remission induction.

## 4 Conclusions

HLH is a rare, yet potentially deadly disorder, which needs to be recognized promptly to be appropriately managed. This case report describes the clinical course of a patient who developed HLH in the setting of an EBV infection, complicated by a massive epistaxis. Early recognition of the disease still remains a challenge, especially while waiting for the paraclinical data, and is the key to rapid treatment instauration.

## Author contributions

**Conceptualization:** Maria Oana Mărginean.

**Data curation:** Mihaela Ioana Chincesan.

**Formal analysis:** Maria Oana Mărginean.

**Investigation:** Maria Oana Mărginean, Eniko Molnar, Mihaela Ioana Chincesan.

**Methodology:** Maria Oana Mărginean, Eniko Molnar, Mihaela Ioana Chincesan.

**Project administration:** Maria Oana Mărginean.

**Supervision:** Maria Oana Mărginean.

**Validation:** Maria Oana Mărginean, Eniko Molnar.

**Writing – review & editing:** Maria Oana Mărginean, Eniko Molnar, Mihaela Ioana Chincesan.
